# Advances in understanding the pathogenesis of post-traumatic epilepsy: a literature review

**DOI:** 10.3389/fneur.2023.1141434

**Published:** 2023-08-10

**Authors:** Mingzhu Fang, Wanyu Liu, Jinmei Tuo, Mei Liu, Fangjing Li, Lijia Zhang, Changyin Yu, Zucai Xu

**Affiliations:** ^1^Department of Neurology, Affiliated Hospital of Zunyi Medical University, Zunyi, China; ^2^Sichuan Provincial People’s Hospital Medical Group Chuantou Xichang Hospital, Xichang, China; ^3^Department of Nursing, Affiliated Hospital of Zunyi Medical University, Zunyi, China; ^4^The Collaborative Innovation Center of Tissue Damage Repair and Regeneration Medicine of Zunyi Medical University, Zunyi, China

**Keywords:** post-traumatic epilepsy, ultrastructural, iron ion, free radicals, hippocampus

## Abstract

Severe head trauma can lead to seizures. Persistent epileptic seizures and their progression are associated with the severity of trauma. Although case reports have revealed that early use of anti-seizure drugs after trauma can prevent epilepsy, clinical case–control studies have failed to confirm this phenomenon. To date, many brain trauma models have been used to study the correlation between post-traumatic seizures and related changes in neural circuit function. According to these studies, neuronal and glial responses are activated immediately after brain trauma, usually leading to significant cell loss in injured brain regions. Over time, long-term changes in neural circuit tissues, especially in the neocortex and hippocampus, lead to an imbalance between excitatory and inhibitory neurotransmission and an increased risk of spontaneous seizures. These changes include alterations in inhibitory interneurons and the formation of new, over-recurrent excitatory synaptic connections. In this study, we review the progress of research related to post-traumatic epilepsy to better understand the mechanisms underlying the initiation and development of post-traumatic seizures and to provide theoretical references for the clinical treatment of post-traumatic seizures.

## 1. Introduction

Annually, approximately 64–74 million people suffer from traumatic brain injury (TBI) worldwide. The severity of TBI varies from mild to severe and may lead to post-traumatic syndromes, including depression and cognitive, emotional, and behavioral defects. TBI can increase the susceptibility to epilepsy and the incidence rate of epilepsy; this phenomenon is called post-traumatic epilepsy (PTE). However, in-depth studies on TBI, its biomarkers, and preventive measures are lacking. Furthermore, the exact mechanisms underlying the epileptic factors that may lead to PTE remain unclear.

Although the type and severity of damage can partially predict susceptibility to PTE, similar damage does not always lead to PTE (1). The location of the lesion may affect the risk of PTE because temporal lobe lesions after TBI are associated with a high incidence of early epilepsy and the longitudinal development of PTE (2). Penetrating lesions in the motor and parietal lobes are associated with an increased risk of PTE (3). Neuroinflammatory reactions occur rapidly. Based on increasing evidence from human and animal studies, neuroinflammatory reactions play a role in the development of TBI (4). Generally, inflammatory factors, chemokines, and complement proteins are rapidly released after TBI. This immune response signal is composed of multiple cellular mediators that initiate acute-phase responses (5–11). Based on these signals, astrocytes and resident microglia are activated, proliferated, and migrated to the site of injury (12, 13). Peripheral immune cells also infiltrate the brain during the TBI response. Once this immune/neuroimmune response is activated to re-establish tissue homeostasis, these immune cells clear the debris and identify potential pathogenic signals. Although the strongest neuroinflammatory reaction occurs relatively early (within hours or days after injury), low levels of neural inflammation tend to persist for a long time (12–17). Acute, early, and chronic neural inflammations are also associated with epilepsy.

Some of the earliest neuropathologies indicated that progressive glial proliferation at the site of brain injury is a major factor in the formation of epileptic foci (3, 18). Increasing evidence supports the presence of glial scars and other neuroinflammatory mechanisms in PTE. Griffin and Mrak provided a platform that led to renewed attention to the mechanisms of neuroinflammation in neuropathological diseases. In this study, we provide a comprehensive review of neuroinflammation after TBI, focusing on the neuroinflammatory mechanisms that promote seizures, epilepsy, and PTE development. We also reviewed the experimental animal models used in PTE research, including those with neurophysiological and structural abnormalities, which are considered the basis for the increased tendency of spontaneous seizures in the injured brain. Notably, we focused on the modification of the damaged dentate gyrus synaptic network, which is related to the occurrence of PTE. The hippocampus is an important structural region involved in epilepsy. The dentate gyrus is particularly vulnerable and usually undergoes time-dependent structural reorganization. It is a widely used model system for changes in the synaptic circuits of cortical structures in epilepsy and is one of the best regions for characterizing changes in structure–function correlations after TBI.

Therefore, this review aimed to understand the mechanisms underlying epilepsy after TBI and to identify, develop, and validate therapeutic strategies to prevent PTE.

## 2. Epidemiological characteristics of PTE

Epilepsy is estimated to affect approximately 0.5–2% of the population. For patients who experienced a sudden head injury and/or were hospitalized for TBI, the incidence rate increased to approximately 5–7% (1, 18, 19). The higher the severity of the injury, the higher the risk of PTE, which is 10-fold higher in military patients with penetrating head trauma. In extreme cases, the incidence of PTE after a severe penetrating head injury is greater than 50% (20–22). Overall, up to 20% of symptomatic epilepsy cases are estimated to be caused by TBI, which is the most commonly known cause of seizures and epilepsy.

Although typical antiepileptic drugs such as levetiracetam and phenytoin sodium can effectively control early seizures that occur within the first week of TBI (23), these treatments do not necessarily improve the risk of PTE (24, 25). Approximately one-third of patients with PTE are resistant to antiepileptic therapy (25–27). Furthermore, the side effects of antiepileptic drugs are more severe in patients with PTE (28). Therefore, understanding the pre-epileptic mechanisms of TBI is crucial for diagnosing and treating PTE and improving the quality of life of these patients.

## 3. Brain structure and network-related mechanisms

Epilepsy is caused by an imbalance between excitation and inhibition in the central nervous system. Accordingly, factors that can increase the excitability or decrease the inhibition of the nervous system may induce epilepsy. The occurrence of PTE is no exception. Many studies have shown that axonal sprouting and the formation of new excitatory synaptic connections are key to the occurrence of PTE. In a pyramidal cell model (the classic PTE model), axon terminals emerged in the injured area, and GAP43, a protein related to axon terminals, was reactivated. On day three after injury, the expression of neurofilament proteins in the interneurons and vertebral cells began to increase and lasted for several weeks. Two to three weeks after injury, the frequency of excitatory postsynaptic currents in the cortical V-layer vertebral cells increases significantly and is accompanied by axon growth, axon branching, and an increased number of synapses (29–32). Using free glutamate laser scanning light stimulation technology, synaptic connections in the cortical disconnection model were observed. The number of excitatory connections of pyramidal cells in the epileptogenic focus and the amplitude of excitatory postsynaptic potentials recorded by electrophysiology increase significantly (33). These results confirm that the axon sprouting observed in the anatomy is new and functional and can be repeatedly overexcited to produce epileptiform discharges. Therefore, the enhancement of excitatory synapses plays an important role in the occurrence of PTE.

One of the most common causes of epilepsy after brain trauma is the formation of cortical scars (34, 35). The normal cortex was rich in the capillary network and leptomeningeal artery, whereas the middle area between the scar and normal cortex represented the anastomosis of the soft and dural arteries lacking capillaries. Damage to the myelin sheath and neurons and regeneration of neuronal synapses were also observed. The blood flow in the middle area is only 1/50 of that in normal brain tissue. Progressive gray matter atrophy, glial hyperplasia, and scar formation caused by ischemia pull surrounding tissues toward the center of the scar. Furthermore, vascular pulsation induces mechanical tension on the dendrites of the neurons in the middle area. Dendrites are highly sensitive to this tension, which causes the middle area to become the epileptic focus. Focal damage to the blood–brain barrier (BBB) is an initial factor in early post-traumatic seizures.

Both primary and secondary brain injuries caused by brain trauma can cause changes in the neurons themselves and their surrounding vascular tissues and glial cells and promote the excessive discharge of neuronal supersynchronization. The axons of normal neurons send out branches that inhibit the excitability of the neurons through a feedback circuit composed of interneurons. Collateral inhibition is most significant when excitability is abnormally elevated. When brain trauma occurs, these collaterals are first affected and lose their inhibitory effect, resulting in excessive excitation of neurons and triggering seizures (36). In addition, injury to superficial cortical dendrites during brain trauma can cause continuous depolarization and discharge of local neurons, stimulate downstream neurons, and induce abnormal excitation, which together play a role in promoting seizures.

The term “epilepsy” refers to recurrent or unprovoked seizures. This process usually involves structural changes in the neural circuit owing to gradual neuronal damage and the “self-repair” mechanism, which develops during latency at different times, eventually ending in spontaneous, recurrent seizures. This process also has a variable latency period, indicating that a series of gradual cellular changes may be involved. Therefore, post-traumatic seizures in humans are usually classified according to the time after injury as immediate or impact-related (24 h after injury), early (1 week after injury), and late (1 week after injury). This classification model represents different pathophysiological processes. Understanding the epileptogenic process after TBI will help to clarify the importance of these cellular mechanisms in PTE and promote the development of new therapeutic targets. Trauma initiates a multidimensional cascade of cellular and molecular events with three types of overlapping responses in the brain: primary and secondary injuries and “self-repair” mechanisms. An important goal of studying the occurrence of PTE is to separate damage-induced cellular changes that promote epilepsy from compensatory and “self-repair” responses.

**Figure 1:**
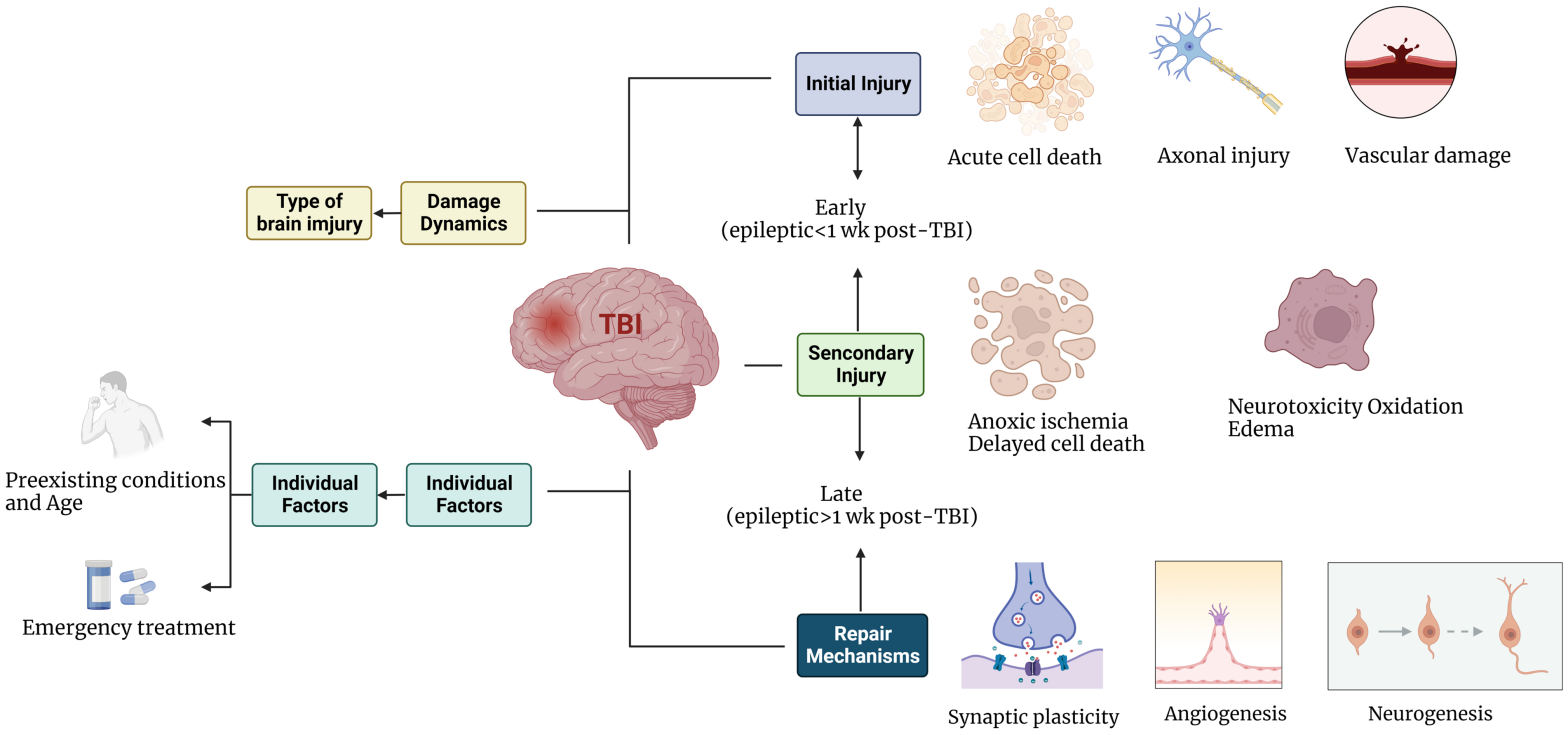
Progress of cellular events after brain trauma.

As shown in Figure 1, a series of cellular and molecular events after TBI involves three types of time-overlapping reactions in the brain: primary and secondary injury and the “self-repair” mechanism. In a multimodal magnetic resonance imaging (MRI) neuroimaging study evaluating brain trauma, PTE, and depression, the value of early quantitative MRI measurements of TBI in predicting traumatic epilepsy was explored. The degree of damage to the cortex, ipsilateral hippocampus, thalamus, and contralateral hippocampus in early TBI was found to be related to the occurrence of traumatic epilepsy, which does not occur immediately after trauma, but may occur within 3 weeks to 2 years after trauma. MRI includes susceptibility-weighted imaging (SWI) and diffusional kurtosis imaging (DKI) for the evaluation of microstructural changes in softening lesions and surrounding tissues after mild and moderate trauma and the possibility of epilepsy. SWI detects intracranial microbleeds with greater sensitivity, which is of great value for evaluating patients with mild brain injuries. Microbleeds in the frontal, parietal, and temporal lobes are associated with post-traumatic depression (25). Currently, it is difficult to determine whether depression during the post-traumatic rehabilitation stage is related to trauma. However, after traumatic epilepsy, the probability of depression in patients with epilepsy is relatively high, with epilepsy and associated depression affecting each other.

Magnetic resonance elastography (MRE) scans with an MRI pulse train to generate propagated sound waves and measurable tissue displacement are considered a particularly sensitive imaging tool that could increase the potential for early diagnosis of neurological diseases. It has been applied to many neurological diseases, such as multiple sclerosis, Alzheimer’s disease, frontotemporal dementia, Parkinson’s disease, and amyotrophic lateral sclerosis. Regarding preclinical models of MRE, several studies have indicated its potential use in TBI. Using a TBI mouse model, longitudinal MRE studies showed that the elastic modulus of the injured brain tissue was higher than that of the contralateral hemisphere 1 h after injury. However, the elastic modulus decreased 1 day after injury and recovered in the brain tissue close to the contralateral hemisphere 7 days later. Although more preclinical and clinical validation is needed for this emerging model in the future, MRE technology holds promise for measuring the mechanical properties of the brain *in vivo* through noninvasive scans, with the potential to improve prognosis (37).

In humans, primary damage to the meninges and vasculature has been observed in the absence of significant brain damage after minor head trauma, as part of an ongoing mild TBI study evaluating MRI comparison studies of patients with mild craniocerebral injury who arrived at the emergency room within 48 h (38). Over 30 months, 142 patients with a baseline Glasgow coma score of 15 with significant loss of consciousness or post-traumatic memory but no substantial injury on clinical computed tomography (CT) were enrolled. CT showed meningeal hemorrhage in 18 patients (12.7%), including subarachnoid hemorrhage in 13 patients (9.1%) and subdural hemorrhage in seven patients (4.9%). Focal enhancement of the meninges was observed on FLAIR MRI contrast imaging with no accompanying meningeal hemorrhage in 53 (36.9%) of 69 (48.6%) patients. This enhancement is the result of gadolinium contrast agent extravasation into spaces containing free fluids with a T1 relaxation time constant equal to the relaxation time of cerebrospinal fluid (CSF).

## 4. Epileptogenic mechanisms of inflammatory reaction

Epilepsy pathogenesis is closely associated with immune regulation. Immune cytokines play a key role in immune regulation and epilepsy. The inflammatory factors released by damaged neurons and glial cells initiate a vicious cycle of brain inflammation. Brain injury leads to the early activation of microglia and stimulates the production of cytokines and the expression of damage-associated molecular pattern-related receptors on the surface, leading to neuronal excitability. Molecules released by active glial cells and neurons increase the hyperexcitability of cells, and blood cells tend to migrate to the brain. Damage to the BBB leads to the infiltration of immune cells into the brain by blood-released proinflammatory factors and promotes excitotoxicity. Subsequently, the activated microglia release signals that bind to astrocyte receptors. Serum albumin-induced astrocyte activation can damage potassium and glutamate transporters. In addition, activated astrocytes release proinflammatory factors that aggravate neuronal hyperexcitability. These factors, either individually or in combination, can cause neurodegeneration. Cytokine-mediated crosstalk between astrocytes, microglia, and glial cells can induce neuronal overexcitation and death. In contrast, neuronal molecules such as heat shock protein (HSP60), high-mobility group box 1 (HMGB1), differentiation cluster 14 (CD14), chemokine (C-X3-C motif) ligand 1 (CX3CL1), and adenosine 5′-triphosphate (ATP) can activate microglia, leading to excitotoxicity. Monocytes and white blood cells release cytokines concurrently, creating an activation cycle that leads to the release of additional inflammatory molecules and triggers a cytokine storm in the brain. Macrophages release major histocompatibility complex II, complementary proteins, proinflammatory interleukins, inducible nitric oxide synthase (iNOS), transcription factors, microRNAs, and other molecules that cause neuronal excitotoxicity. These molecules promote neurodegeneration, initiate neuroinflammatory pathways, and reduce the seizure threshold. HSP60, heat shock protein; HMGB1, high-mobility group box 1; CD14, differentiation cluster 14; CX3CL1, chemokine (C-X3-C motif) ligand 1; ATP, adenosine 5′-triphosphate; inducible nitric oxide synthase. The BBB is typically damaged during brain trauma. One study found that the BBB was damaged in 82.4% of patients with epileptic seizures after brain trauma, which was 25% higher than that in patients without epileptic seizures. The abnormal EEG activity sites recorded in patients with PTE are usually consistent with brain regions with significant BBB damage. Moreover, continuously increased BBB permeability may be related to an increased frequency and degree of seizures in patients with PTE (39). Focal damage to the BBB is the initial factor in early post-traumatic seizures, followed by an abnormal excitability pattern in peripheral nerve seizures. Under normal circumstances, the BBB does not allow most blood-borne proteins from serum to enter the brain. Serum albumin is the most abundant protein in the blood. Normal individuals have low albumin levels in the CSF. After BBB damage caused by TBI, a sudden and continuous increase in albumin levels in the brain parenchyma can lead to seizures (40).

One study found that IL-1β levels are significantly increased in the CSF and serum of patients with TBI, which is related to the incidence of PTE (32). Recurrent seizures can lead to peripheral and central inflammation. Microglia and astrocytes release IL-1β during seizures and activate IL-1β receptor/Toll-like receptor signaling. Epilepsy induces IL-1 β release. The activation of adrenergic acids leads to many interrelated damage processes, including BBB damage. Further upregulation of signal transduction leads to an increase in extracellular potassium and glutamate levels, a decrease in the seizure threshold, and the loss of neurons. A peripheral inflammatory reaction appears in the center, leading to hyperfunctioning of the hypothalamus–pituitary–adrenal axis. Upon the activation of dioxygenase, the levels of toxic metabolites in the tryptophan metabolic pathway increase. Upon activation of the oxidation/nitrification stress pathway, oxygen and nitrogen free radicals increased. These reactions interacted with each other. Thereafter, the inflammation persists, effectively blocking any link in the reaction that may prevent the development of epilepsy-related depression (41). Thus, inflammation plays an important role in epilepsy. It can improve depression in patients with epilepsy by controlling inflammatory reactions.

## 5. Mechanisms underlying imbalances in neurotransmitter regulation

### 5.1. 5-HT

5-HT is a neurotransmitter that plays a role in central nervous system (CNS) infections and the peripheral nervous system, and several non-neuronal tissues are involved in membrane receptor interactions. The 5-HT system is also involved in the pathogenesis of epilepsy and neuropathic pain. The 5-HT1A, 5-HT2C, 5-HT3, and 5-HT7 receptors are involved in the occurrence and maintenance of epilepsy and changes in susceptibility to epilepsy. However, a 5-HT deficiency can cause seizures. Some antiepileptic drugs exert antiepileptic effects by increasing the levels of extracellular 5-HT receptors. The lack of 5-HT2 receptors significantly increases the risk of seizures. 5-HT2 receptors directly or indirectly control the excitability of neurons in the entire network structure by interacting with the monoamine neurotransmitters γ-aminobutyric acid (GABA) and glutamate (Glu) (42).

### 5.2. Glu

Glutamate is an excitatory neurotransmitter. Glu in and out of cells maintains a balance through the transport of protein channels between neurons and glial cells. After brain trauma, the extracellular Glu levels in the neocortex increase rapidly (43). Cell damage and excessive synaptic activity after injury may increase the release of Glu from neurons and glial cells and reduce their reuptake. Thereafter, GLUergic receptors may become overexcited, eventually leading to neuronal damage or death, which is known as excitotoxicity (44, 45). Excitotoxicity is a major cause of various pathological changes following brain trauma. Brain trauma is a stress factor that can directly enhance the activity of glycinergic receptor channels and further aggravate the excitatory toxicity of brain tissue. A long-term imbalance in Glu levels will continue to affect the excitability of neurons, disrupt the balance between excitability and inhibition of the nervous system, and lead to epilepsy.

During seizures in patients with epilepsy, the expression of phospho-activated glutaminase increases in the hippocampus, converting glutamine to glutamic acid and thereby destroying the balance of glutamic acid in the body. Furthermore, the activity of glutamate dehydrogenase, which removes glutamic acid, decreases, leading to a decrease in glutamic acid degradation. Therefore, a disorder in glutamate metabolism in patients with epilepsy leads to increased glutamate synthesis and decreased glutamate degradation, which is related to intractable epilepsy (46).

In TBI, injured nerve cells consume energy and release glutamate and potassium ions from glial cells into the extracellular fluid, further aggravating the brain injury. Numerous studies have shown that Glu plays an important role in the pathophysiology of epilepsy and depression. First, the rapid metabolism of glutamate ensures that the postsynaptic target cells receive immediate stimulation to enable precise regulation. Second, high concentrations of extracellular Glu are associated with neurotoxicity. Therefore, the balance of glutamate function in the gap must be emphasized. At present, several lines of evidence, such as dysfunction of the glutamate transporter, abnormal concentrations of glutamate and GABA in the cerebral cortex, and the antidepressant effects of glutamate receptor antagonists, ultimately support the involvement of glutamate in the pathogenesis of depression (47).

In the study of neurotransmitter receptors related to the pathogenesis of epilepsy, kainate receptors belonging to the glutamic receptor family kainate (KA) are structural analogs of glutamate, an excitatory amino acid naturally extracted from seaweed. In animal models of KA-induced epilepsy, KA induces seizures and neuropathological damage in temporal lobe epilepsy, indicating that it is a potent toxin. The KAR family was identified by KAR subunit cloning and is composed of five receptor subunits: GLUR5, GLUR6, GLUR7, KA1, and KA2. The expression of GLUR5 was higher in patients with refractory epilepsy (48–50). The temporal cortex is also the lesion site of refractory temporal lobe epilepsy, but the main site of action of GLUR5 is not in the temporal cortex but in the hippocampus, the core lesion site of temporal lobe epilepsy. The hippocampus has been the dominant site for studying GLUR5. Our results further confirm that GLUR5 is involved in the pathogenesis of refractory temporal lobe epilepsy by acting on the hippocampus (51). High GLUR5 expression in patients with refractory epilepsy suggests its involvement in the pathogenesis of refractory epilepsy. Topiramate is the only antiepileptic drug that blocks the GLUR5 receptor. Topiramate has multiple mechanisms for blocking sodium channels, increasing GABA-mediated inhibition, blocking glutamate-mediated nerve excitability, and affecting chloride membrane transport and calcium channel blockade. Thus, it may act as an antiepileptic drug by selectively acting on GLUR5 receptors. It is necessary to further clarify the pathogenic relationship between GLUR5 and human refractory temporal lobe epilepsy and its pathological mechanism in refractory temporal lobe epilepsy, to provide a possible theoretical basis for the development of new antiepileptic drugs (52).

### 5.3. γ-aminobutyric acid

GABA is the main inhibitory neurotransmitter in the CNS, and its release from interneurons plays a role in regulating excitatory neurotransmission. Glutamine in astrocytes is transported to GABAergic neurons for conversion to glutamate, which is then immediately converted to GABA by glutamic acid decarboxylase. Glutamate, glutamine, and GABA depend on intermediates in the tricarboxylic acid cycle. Therefore, defects and inefficiencies in cellular energy metabolism, such as impaired tissue perfusion and increased neuronal metabolic demands after TBI, also lead to inadequate transmitter production. GABA is stored in presynaptic vesicles and released to postsynaptic terminals that may be located in dendritic processes, cell bodies, axons, or other axon terminals (53).

Upon release, GABA acts on the GABA-A and GABA-B receptors. GABA-A receptors are postsynaptic ion receptors containing at least 16 subunits. Different subtype combinations exhibit different physiological and pharmacological features. The combination of these isoforms depends on their colocalization at the neuronal membrane (54). The GABA-A subunits that have received the most attention in TBI studies are α1, γ2, α4, and δ1. These subunits contribute to the ability of GABA interneurons to modulate neuronal signaling through phase and tonic inhibition. These two inhibitors exhibit unique functions. Phasin inhibition reduces the excessive excitability of postsynaptic cells and plays an important role in the generation and regulation of θ and γ oscillations. Alternatively, tonic inhibition could more consistently maintain the amount and duration of postsynaptic depolarization (55).

Postsynaptic inhibition mediated by GABA receptors plays an important role in normal brain function and epilepsy. Blocking GABAergic neurotransmission with drugs can induce acute epileptic discharges and seizures, indicating that disinhibition is a key factor in seizures. In different PTE models and related studies on patients with secondary epilepsy after brain trauma, GABAergic inhibition was weakened, including the loss of inhibitory interneurons, changes in connectivity and anatomical structure, and abnormalities in the postsynaptic GABA receptor (56–58).

TBI is a major cause of death and is typically associated with PTE. TBI causes distinct changes in the neuronal circuits, leading to an imbalance between cortical excitation and inhibition. The change in neurotransmitter concentration was found to be significantly related to the receptor population and cell survival. Subsequent cellular changes may trigger PTE. Changes in the GABA receptor subunit mRNA levels in the thalamic cortex may be related to neuronal degeneration. Although changes in neurotransmitters in PTE have been studied, the specific potential molecular pathways have not been fully elucidated. Astrocyte changes induced by brain injury may impair GABA-mediated neurotransmission, leading to epilepsy. Accordingly, assessing the role of neurotransmitters, especially GABA, in the changes in acute neurons and astrocytes during TBI may provide a new target for the treatment of PTE (59).

### 5.4. Calcium

The excessive influx of calcium ions (Ca^2+^) plays an important role in the occurrence of epilepsy. At the cellular level, epilepsy is caused by the excessive spontaneous discharge of neurons, which is based on cell depolarization caused by transient Ca^2+^ influx. A study performed using the PTE model revealed abnormalities in the Ca^2+^ channel on the presynaptic GABAergic terminals acting on the V-layer vertebral cells in the cortex, which led to a weakening of the inhibitory effect of the nervous system (60). The rapidly increasing levels of extracellular Glu after brain injury play a role in the Ca^2+^ channels. Glu binds to the N-methyl-d-aspartate receptor and activates receptor-operated calcium channels, which can cause Ca^2+^ influx. When Glu binds to KA and QA receptors, it activates Na^+^ channels and depolarizes the cell membrane. When the membrane potential reaches a certain level, the voltage-dependent calcium channel is activated and opened, further intensifying the extracellular Ca^2+^ influx. Excessive Ca^2+^ influx caused by multiple channels eventually leads to the epileptic discharge of neurons (61). After brain injury, the regulatory mechanism of K^+^ is also abnormal, leading to an increase in the extracellular K^+^ concentration, which can promote the transformation of non-explosive neurons into explosive neurons and carry out cluster over discharge, leading to excessive excitation and synchronization of neurons, ultimately causing seizures (62).

### 5.5. Aquaporin-4

TBI is the most common cause of death and disability among young people. Brain edema is a serious complication of brain trauma, and its occurrence is related to changes in aquaporin (AQP) expression. The most important aquaporin protein in the brain, aquaporin-4 (AQP4), is mainly expressed on astrocyte membranes, and its distribution is extremely uneven. AQP4 is expressed in the endfeet of astrocytes, which are adjacent to blood vessels and the subpial and periventricular regions. This is known as the “polar distribution” of AQP4 and is closely related to water transport. It mainly regulates the bidirectional flow of water in the blood and CSF to maintain ion concentration (63–67). A decrease in AQP-4 can lead to ion regulation dysfunction and epilepsy. Loss of AQP-4 expression is accompanied by a decrease in astrocyte and hippocampal nerve cell regeneration, which can aggravate depressive symptoms (68).

### 5.6. Oxidative stress

Emerging evidence suggests that acute seizures can cause oxidative stress. On the one hand, due to increased oxygen consumption and reduced antioxidant defenses, neuronal cells are highly susceptible to oxidative damage, and neuronal cells contain large amounts of polyunsaturated fats, which are easily oxidized. On the other hand, the degradation of superoxide dismutase promotes highly reactive peroxynitrite (ONOO^−^), a powerful oxidizing agent that can lead to increased ROS production, the oxidation of DNA, proteins, and lipids, and the loss of ion channel dysfunction, leading to brain damage. Excessive production of free radicals can disrupt intracellular calcium homeostasis, thus affecting neuronal excitability. Therefore, antioxidant therapy may be an important technique for reversing neurodegenerative processes associated with epilepsy (69).

### 5.7. Other mechanisms

Several studies have redefined the classical role of astrocytes in the brain. Astrocytes were initially considered the main supporting cells (70), functioning as neurons and helping maintain brain homeostasis. Despite early recognition of the role of astrocytes in the injury response (71), their extensive role in the pathogenesis of the inflammatory response continues to be recognized and explored (72). Therefore, astrocytes are not only classified as supporting cells but also as cells that actively and directly participate in many aspects of neural function. Astrocytes are the most abundant cell type in the brain and are involved in the regulation of ion homeostasis, maintenance of the BBB, metabolism of neurotransmitters, and the provision of nutrition and energy support for neuronal function (73). Astrocytes are key components of learning, memory, sleep, and other basic brain functions (5, 23) and play an important role in neuroinflammatory responses.

Astrocytes play a key role in regulating neuronal activity by exchanging neuronal pyruvate for lactic acid to activate neuronal metabolism and increase neuronal NADH levels (7). Astrocytes are also involved in processing neuronal information. Their processing activity involves thousands of synapses and controls neuronal activity through neurotransmitter uptake and release (74). Astrocytes also regulate Glu and GABA availability in the synaptic space, thereby regulating synaptic transmission (25–27). Accordingly, astrocytes may promote PTE via different mechanisms. In the present study, we focused on the mechanisms underlying neuroinflammation. Astrocyte activation is a major cellular component of neuroinflammatory reactions, and glial cell proliferation is common after TBI. Astrocytosis can occur as part of the neuroinflammatory response. In the postmortem TBI human brain, extensive astrocytosis occurs at the main injury site and in the ipsilateral and contralateral brain regions away from the initial injury site (14, 15, 17). Notably, it is impossible to distinguish glial cell proliferation induced by epilepsy from glial cell proliferation alone, which may induce epilepsy in the brains of patients with postmortem epilepsy. Similar glial cell proliferation patterns have been observed in many TBI animal models (3, 7, 10, 18, 19, 40, 75), enabling studies on potential astrocytic mechanisms of epilepsy. The response of astrocytes to TBI involves the death and axonal degeneration of neurons and the rapid release of inflammatory complement system factors, cytokines, and chemokines from microglia, neurons, and astrocytes. Microglia, astrocytes, and neurons are activated and altered following TBI. Activated microglia secrete cytokines such as interleukin-1 α, tumor necrosis factor, and complement component 1 subcomponent q, which can induce the A1 astrocyte phenotype. Astrocytes suffer from gap junction decoupling, synaptic neurotransmitter clearance, and damage to the metabolic cycle. The cytokines interleukin-6, interleukin-1β, transforming growth factor β, and chemokine CCL2 produce a neuroinflammatory environment at high concentrations. Regardless of the source, the release of this cytokine may affect the pathological functions of astrocytes, particularly those involved in physiological signal transduction and epilepsy.

## 6. Treatment of PTE

Treatment of early post-traumatic seizures does not affect the incidence of post-traumatic seizures. Conventional prophylactic anticonvulsants are not used in patients with head injuries, and treatment in the acute phase does not reduce mortality or disability. A large number of studies have shown that there are different ways to treat PTE, mainly drug therapy and non-drug therapy. Currently, drug therapy is dominant, and non-drug therapies have shown some benefit in the laboratory but have not been established to have any clinical benefit. It has been reported that some drugs such as rapamycin, carbamazepine, phenytoin sodium, sedative-hypnotics, levetiracetam, atorvastatin, losartan, curcumin, and lipoic acid can show antiepileptic effects. In addition, gene therapy, stem cell therapy, and deep brain stimulation have been shown to inhibit the onset and progression of seizures. However, antiepileptic drugs (AEDs) are effective in suppressing epilepsy only if treatment is continued and blood concentrations remain high enough. In addition, long-term treatment with AEDs can produce unwanted side effects, such as chronic cognitive, memory, and behavioral changes, severe hepatotoxicity and neurotoxicity, as well as teratogenic effects and withdrawal effects (76).

## 7. Discussion

Excitotoxicity, neuroinflammation, oxidative stress, and neurodegeneration are the primary pathological mechanisms underlying PTE. TBI is known to initiate a cycle of neuroinflammatory events that trigger oxidative stress reactions, inducing a cascade of events and cycles that aggravate the acute phase and lead to chronic diseases. This review aimed to understand the mechanisms underlying epilepsy after TBI and to identify, develop, and validate therapeutic strategies to prevent PTE. Accordingly, several key conclusions were drawn: (1) the main sources of cell excitotoxicity after TBI include an increased level of extracellular glutamate, an increase in immune cell infiltration, and crosstalk between glial cells and neurons, which are mainly regulated by the network of cytokines and chemokines; (2) the initial immune response to injury is beneficial, stabilizing the imbalance in the system; (3) overproduction of ROS/RNS leads to mitochondrial damage, which is a continuous process during epilepsy and is related to inflammation and neurodegeneration; (4) proinflammatory cytokines and chemokines are key factors that invade blood cells, microglia, astrocytes, and neurons; (5) long-term excessive production of cytokines, lipids, and chemokines triggers cell death; and (6) leukocyte invasion and activation of microglia leads to late tissue damage.

PTE exhibits phenotypic heterogeneity in humans. Understanding this phenotypic heterogeneity is important for the development of new antiepileptic therapies. Both focal and diffuse mechanisms can cause PTE, and approximately 25–30% of PTE cases are related to medial temporal sclerosis. Although surgery is an alternative therapy, it is not recommended in such cases. Therefore, defibrillators remain the first line of treatment. However, AED is not very effective in the treatment of PTE. Accordingly, it is used to treat symptoms but does not improve the underlying condition. Patients taking antiepileptic drugs (AEDs) typically require long-term treatment. However, some patients experienced serious adverse effects over time. Uncontrolled seizures can lead to increased mortality, a decreased quality of life, comorbidities, and depression. Although many antiepileptic drugs are available, progress has not been made in preventing new seizures. Additionally, the reuse of antiepileptic drugs to prevent seizures has been unsuccessful. Understanding epilepsy as a network disorder requires identifying its early stages before the imbalance spreads to other regions of the brain. However, this identification is not simple because many candidate compounds have multiple functions and target multiple pathways. These approaches may also differ between human and animal models, reducing the translational significance of animal models and emphasizing the importance of appropriate time intervals, dosages, targets, and the design of experiments. Goals must be defined based on different injury mechanisms, and different treatment methods must be adopted at specific time points. Finally, a clear understanding of the molecular mechanisms underlying epilepsy will aid in the development of novel therapeutic targets for PTE.

In this review, we summarize the relevant research on the pathogenesis of PTE. These mechanisms are interrelated and do not exist independently. At present, the relevant mechanisms of PTE have not been clarified, and further research is required. It is the most common form of symptomatic epilepsy. Herein, we describe the inflammatory components of the cellular and molecular mechanisms of the CNS that can promote the development of epilepsy after brain trauma. Although the innate inflammatory response to injury is relatively stable, the comprehensive effect leading to PTE is highly variable and depends on time, location, and individual immune responses. Therefore, diagnostic tools that consider multimodal variables must be developed. Furthermore, the adaptive immune components that play a role in the development of PTE must be recognized. However, these variables require further investigation. As PTE is difficult to treat and is more resistant to first- and second-line antiepileptic treatments, the treatment of specific inflammatory components after TBI is expected to eventually produce meaningful diagnostic tools and effective treatment strategies. Therefore, one of the preconditions for preventing PTE is avoiding TBI as much as possible. Once the relevant mechanisms are revealed, better prevention and treatment strategies for PTE can be developed.

## Author contributions

MF, WL, and JT: original draft, design, and methodology. LZ and FL: conceptualization and investigation. JT and ML: supervision and conceptualization. CY and ZX: conceptualization. All authors contributed to the article and approved the submitted version.

## Funding

This work was supported by grants from the Guizhou epilepsy basic and clinical research scientific and technological innovation talent team project (No: CXTD[2022]013), the Collaborative Innovation Center of Chinese Ministry of Education (No: 2020-39), the Guizhou provincial “hundred” level innovative talents funds (No: GCC-2022-038-1), the Guizhou Provincial Science and Technology Foundation (No: ZK2022-656), and the Zunyi City Science and Technology Foundation (No: 2019-71 and 2021-30), the United Foundation of Zunyi City (grant no. Zunshikehe HZ Zi [2021]14), the Lingnan Neurology clinical research project for young and middle-aged doctors of Guangdong Medical Association (grant no. Yueyihui [2020]398).

## Conflict of interest

The authors declare that this study was conducted in the absence of commercial or financial relationships that is construed as potential conflicts of interest.

## Publisher’s note

All claims expressed in this article are solely those of the authors and do not necessarily represent those of their affiliated organizations, or those of the publisher, the editors and the reviewers. Any product that may be evaluated in this article, or claim that may be made by its manufacturer, is not guaranteed or endorsed by the publisher.
